# Differential radiosensitisation by ZD1839 (Iressa), a highly selective epidermal growth factor receptor tyrosine kinase inhibitor in two related bladder cancer cell lines

**DOI:** 10.1038/sj.bjc.6602299

**Published:** 2004-12-21

**Authors:** S B Maddineni, V K Sangar, J H Hendry, G P Margison, N W Clarke

**Affiliations:** 1 Cancer Research-UK Carcinogenesis Group, Paterson Institute for Cancer Research, Wilmslow Road, Manchester M20 4BX, UK; 2 Cancer Research-UK Experimental Radiation Oncology Group, Paterson Institute for Cancer Research, Manchester M20 4BX, UK, Wilmslow Road, Manchester, M20 4BX, UK; 3 Christie Hospital NHS Trust, Wilmslow Road, Manchester M20 4BX, UK

**Keywords:** bladder, radiation, radiosensitisation, ZD1839

## Abstract

The epidermal growth factor receptor (EGFR) is expressed in a wide variety of epithelial tumours including carcinoma of the bladder. Stimulation of the EGFR pathway is blocked by ZD1839 (Iressa), a highly selective EGFR tyrosine kinase inhibitor. Radical radiotherapy is an established organ sparing treatment option for muscle invasive bladder cancer and this study has explored the possibility for the use of ZD1839 as a radiosensitiser in this scenario. The effect of combination treatment with ZD1839 (0.01 *μ*M) and ionising radiation in the established bladder cancer cell lines MGH-U1 and its radiosensitive mutant clone S40b was measured by clonogenic assays. A highly significant radiosensitising effect was seen in both cell lines (*P*<0.001 for MGH-U1 and S40b cell lines). This effect was independent of the concentration of the drug and the duration of exposure prior to treatment with ionising radiation. Cell cycle kinetics of both cell lines was not significantly altered with ZD1839 (0.01 *μ*M) as a single agent. A modest induction of apoptosis was observed with ZD1839 (0.01 *μ*M) as a single agent, but a marked induction was observed with the combination treatment of ZD1839 and ionising radiation. These results suggest a potentially important role for ZD1839 in combination with radiotherapy in the treatment of muscle invasive bladder cancer.

Carcinoma of the bladder is the second most common malignancy of the genitourinary tract accounting for 13 600 new cases in the UK in 1999 (Quinn *et al*, 1999) and an estimated 132 000 directly related deaths worldwide in 2000 (Ferlay *et al*, 2000). Radical radiotherapy is an established organ preserving treatment option for muscle invasive disease with an overall 5-year survival comparable to that of radical surgery in some cases (Shelley *et al*, 2001). There are continuing improvements in the accuracy of delivered radiotherapeutic dose and reductions of target volume irradiated (Zelefsky *et al*, 1998). The use of radiosensitising agents has also been explored in an attempt to improve efficacy and minimise toxicity (Hennequin and Favaudon, 2002).

The epidermal growth factor receptor (EGFR) is one of a family of four closely related transmembrane glycoprotein receptors consisting of an extracellular ligand binding domain, a transmembrane domain and an intracellular tyrosine kinase domain. The receptor exists as an inactive monomer that undergoes homo- or heterodimerisation after activation by ligands such as epidermal growth factor (EGF) and transforming growth factor-*α* (TGF-*α*). This results in receptor-associated tyrosine kinase activation, which triggers a cascade of downstream signalling pathways resulting in cell cycle progression, proliferation, angiogenesis and cell survival. A wide variety of human tumours including breast, NSCLC, bladder and SCC of the head and neck have been reported as expressing moderate to high levels of EGFR (Salomon *et al*, 1995). Activation of EGFR has been shown to result in accelerated tumour growth and is associated with a poor patient prognosis in many solid tumours (Nicholson *et al*, 2001). It has been suggested that cell survival and repopulation may be enhanced by activation and expression of EGFR and the ligand TGF-*∝* that is induced following irradiation (Schmidt-Ullrich *et al*, 1994, 1996, 1997). In view of the crucial importance of the EGFR-mediated signalling cascade to cancer therapeutics, this receptor has been the focus of intense research.

ZD1839, a synthetic anilinoquinazoline, is an orally active, highly selective EGFR tyrosine kinase inhibitor (EGFR-TKI) that inhibits ligand-stimulated EGFR autophosphorylation and signal transduction pathways implicated in the promotion of cancer cell growth and proliferation (Woodburn, 1999). Treatment of the EGFR signalling cascade with ZD1839 offers the potential to modulate and inhibit the inbuilt pathways that protect against ionising radiation and therefore improve the efficacy of radiation-induced cell death (Ciardiello and Tortora, 2001b). In this study, we examined whether combination treatment with ZD1839 and ionising radiation resulted in increased cell death in two closely related bladder cancer cell lines MGH-U1 and its relatively radiosensitive derivative, S40b (McMillan and Holmes, 1991). The effects of combination treatments on cell cycle kinetics and apoptosis were also investigated.

## MATERIALS AND METHODS

### Chemicals and cell lines

The established human bladder cancer cell line MGH-U1 and its radiosensitive mutant clone S40b were cultured in Hams F12 media with 10% foetal calf serum, 1% glutamine and 1% penicillin and streptomycin. Both cell lines were kindly donated by Professor T McMillan (Lancaster, UK) and Dr S Powell (Boston, USA). ZD1839 (Iressa) was kindly donated by Astra Zeneca Pharmaceuticals (Macclesfield, UK).

### Clonogenic assays with ZD1839

Exponentially growing cells from both cell lines were treated with ZD1839 at final concentrations ranging from 0.01 to 10 *μ*M for 24 h. The drug was removed by washing twice with PBS. The cells were then trypsinised, counted using a Coulter Multisizer II Cell Counter® and plated into 60 mm dishes with 5 ml of media. The dishes were incubated for 10 days at 37°C and 5% CO_2_. The media were aspirated and the plates were stained with 1% crystal violet and surviving colonies with greater than 50 cells per colony were counted.

### Growth inhibition assay

Exponentially growing cells were treated continuously for 6 days with 10 ml of ZD1839 at final concentrations ranging from 0.01 to 1000 *μ*M. The drug was changed every 24 h and a further 10 ml of freshly reconstituted ZD1839 was added. On day 7, the cells were trypsinised and counted using a Coulter Multisizer II Cell Counter®.

### Clonogenic assay with ZD1839 and irradiation

Exponentially growing cells were treated with single doses of radiation (0–10 Gy) in a sealed Caesium-137 gamma-irradiator. The irradiated cells were trypsinised, counted using a Coulter Multisizer II Cell Counter® and plated in 60 mm Petri dishes and incubated as before. The process was repeated for exponentially growing cells treated with ZD1839 at concentrations ranging from 0.01 to 10 *μ*M for 1–6 days prior to irradiation. The drug was replenished daily. Colonies were stained and counted as before. The clonogenic survival data were fitted to a radiation survival curve using the using the linear quadratic equation and the DRFIT Radiation Survival Fitting Program (Roberts, 1990). Differences were assessed using analysis of variance and an *F*-test.

### EGFR expression

The expression of EGFR was determined using an EGFR Elisa Analysis Kit (Oncogene Research Products, San Diego). Exponentially growing cells were treated with a detector antibody to EGFR and incubated at 37°C for 4 h. After washing with buffer supplied with the kit, horseradish peroxidase-conjugated streptavidin was added and after 1 h absorbance was measured using a Plate Reader at 450/595 nm.

### Cell cycle analysis

Cell cycle distribution was analysed at 0, 24 and 48 h after exposure to ZD1839 for 24 h using flow cytometry with propidium iodide (PI) staining. Cells were treated with ZD1839 (0.01–10 *μ*M) for 24 h. The drug was washed off with media twice. For cell cycle distribution analysis at 0 h following exposure to ZD1839 for 24 h, cells were treated as described below. For cell cycle distribution analysis at 24 and 48 h following exposure to ZD1839, 10 ml of media was added and cells were incubated at 37°C for a further 24 and 48 h from the time of exposure to ZD1839. The cells were trypsinised, centrifuged at 1000 r.p.m. for 5 min, then washed with PBS and centrifuged at 2000 r.p.m. for 5 min. Cell pellets were fixed with 500 *μ*l of ice-cold 70% ethanol and quickly vortexed. The cells were centrifuged at 2000 r.p.m. and the supernatant discarded. Ribonuclease A (50 *μ*l of 100 *μ*l ml^−1^ solution) was added and incubated for 5 min. PI stain (300 *μ*l of 50 *μ*g ml^−1^ solution) was added and incubated for 15 min. The specimens were analysed using Flow Cytometry at 620 nm.

### Apoptosis analysis

Apoptosis was evaluated using Annexin V–FITC conjugates and PI staining. Exponentially growing cells were treated with ZD1839 (0.01 *μ*M) for 24 h followed by irradiation (2–10 Gy), or received irradiation alone (2–10 Gy). The cells were trypsinised and centrifuged at 1000 r.p.m. for 5 min. The pellets were washed with 500 *μ*l of cold PBS, centrifuged at 500 **g** for 5 min and 1 ml of binding buffer was added per 10^6^ cells. A measure of 10 *μ*l of 25 *μ*g ml^−1^ Annexin V–FITC and PI stain (10 *μ*l of 50 *μ*g ml^−1^ solution) were added and samples were incubated in the dark for 15 min. The samples were analysed using flow cytometry.

## RESULTS

### Growth inhibition and clonogenic survival

‘The clonogenic survival of both cell lines was not significantly affected by exposure to ZD1839 for 24 h, compared to that of the controls, at the concentrations studied (0.01–10 *μ*M). The length of continuous exposure to ZD1839 (1–6 days) at concentrations of 0.01–10 *μ*M also had no significant effect on cell counts or on clonogenic survival after 10 days (six doubling times) (data not shown).

**Figure 1:**
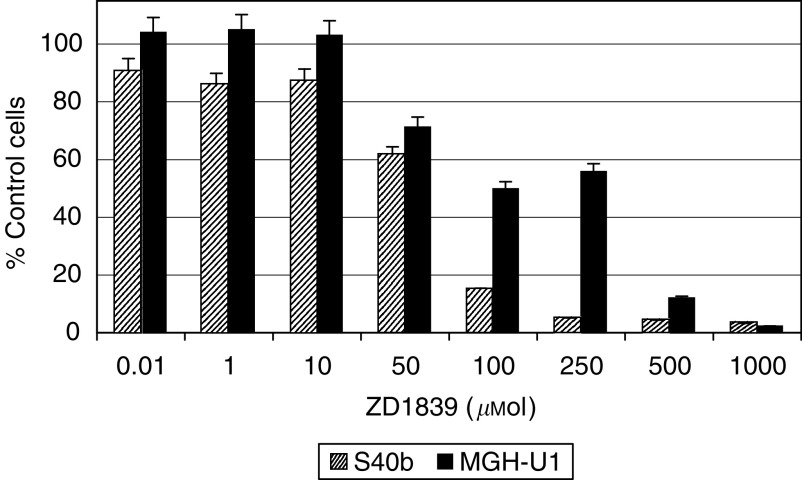
Survival of cells treated with ZD1839 (0.01–1000 *μ*M) for 6 days with drug replenished daily. Cell counts were performed after day 6 of drug exposure. The IC_50_ was 59 *μ*M for S40b and 98 *μ*M for MGH-U1, respectively.

Continuous exposure to ZD1839 at concentrations up to 1000 *μ*M for 6 days with the drug being replenished every 24 h resulted in a significant growth inhibitory effect at high concentrations of the drug. The IC_50_ calculated on cell counts was 98 and 59 *μ*M, respectively, for the MGH-U1 and S40b cell lines, Figure 1.

### ZD 1839 and irradiation

**Figure 2:**
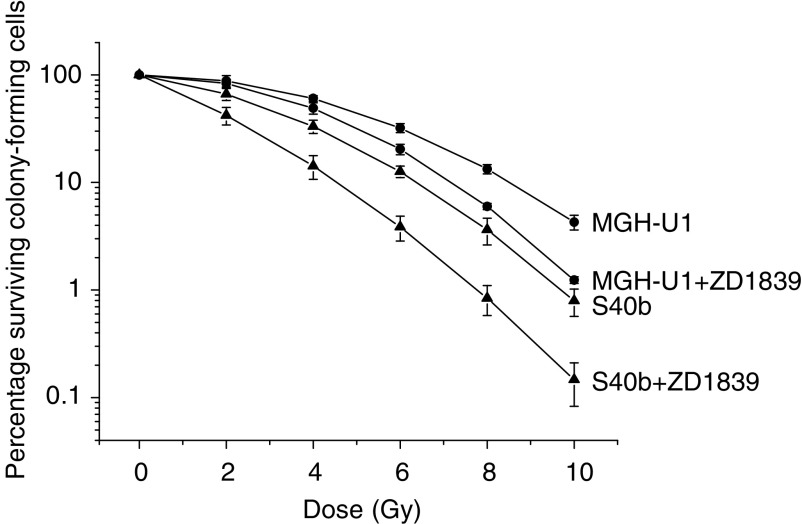
Radiosensitising effect of exposure to ZD1839 for 24 h prior to treatment with ionising radiation. Radiation Survival curves were plotted for the MGH-U1 cell line (•) and the S40b cell line (▴) without ZD1839. Radiation survival curves were plotted for both cell lines following treatment with ZD1839 (0.01 *μ*M) for 24 h prior to irradiation. A highly significant radiosensitising effect was demonstrated for both cell lines treated with ZD1839 over the radiation dose range examined (*P*<0.001 for both cell lines).

**Figure 3:**
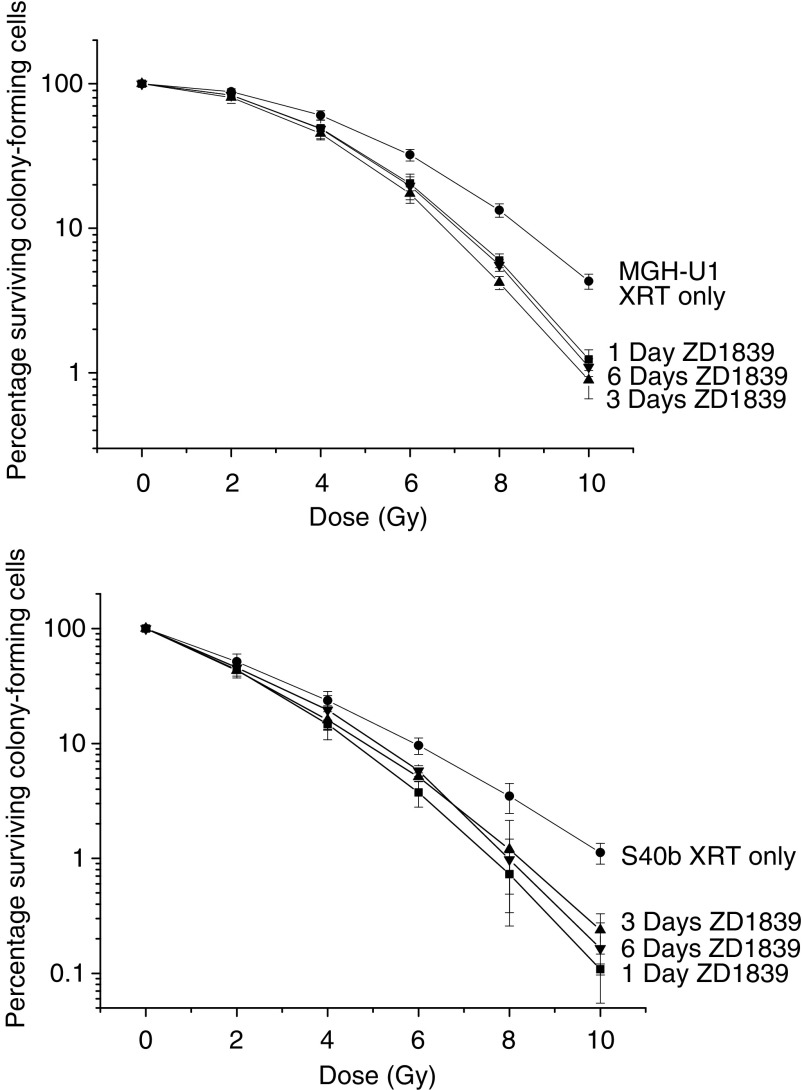
Radiosensitising effect of exposure to ZD1839 for variable periods prior to irradiation on the MGH-U1 and S40b cell lines. Cells were exposed to ZD1839 (0.01 *μ*M) for 1, 3 and 6 days prior to irradiation with the drug being replenished every 24 h. No difference in the radiosensitising effect was detected whether drug was administered for 24 h or 6 days prior to irradiation, although overall radiosensitisation was still highly significant (*P*<0.001). (XRT only=•; ZD1839 1 day=▪; ZD1839 3 days=▴; ZD1839 6 days=▾).

**Figure 4:**
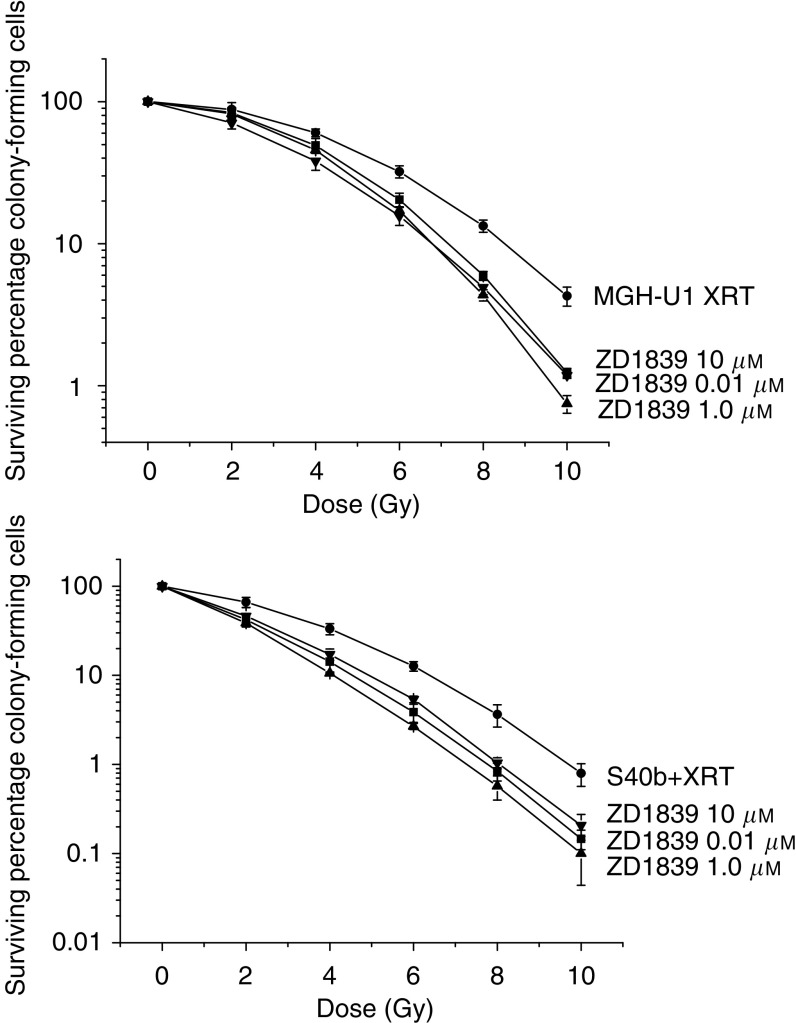
Radiosensitising effect of exposure to increasing concentrations of ZD1839 for 24 h prior to irradiation on the MGH-U1 and S40b cell lines. Cells were exposed to ZD1839 0.01, 1.0 and 10 *μ*M for 24 h prior to irradiation. No significant difference was demonstrated in the radiosensitising effect with the higher concentrations of ZD1839 compared to ZD1839 at a concentration of 0.01 *μ*M (*P*=0.8), although overall radiosensitisation was still highly significant (*P*<0.001). (XRT only=•; ZD1839 0.01 *μ*M=▪; ZD1839 1.0 *μ*M=▴; ZD1839 10 *μ*M=▾).

ZD1839 demonstrated a significant radiosensitising effect on both the radiosensitive S40b cell line (*P*<0.001) and the radioresistant MGH-U1 cell line (*P*<0.001). For MGH-U1, the survival fraction at 2 Gy (SF_2_) was 0.88 and at 10 Gy (SF_10_) was 0.042. For S40b, the SF_2_ was 0.66 and the SF_10_ was 0.0079. The SF_2_ for the combination exposure to ZD1839 for 24 h preirradiation at a concentration of 0.01 *μ*M was 0.83 and 0.42 for MGH-U1 and S40b, respectively. The SF_10_ for the combination exposure to ZD1839 for 24 h preirradiation at a concentration of 0.01 *μ*M was 0.012 and 0.00146 for MGH-U1 and S40b respectively, Figure 2. The radiosensitising effect of ZD1839 on cell kill was found to be more pronounced at higher radiation doses as demonstrated by the SF_10_ values. Prolonged exposure to ZD1839 for 3 and 6 days preirradiation with the drug being replenished daily did not improve the overall radiosensitising effect of the 24 h exposure regimen. In the MGH-U1 cell line, the SF_2_ for 3 and 6 days exposure was 0.80 and 0.83 and the SF_10_ was 0.0089 and 0.0109, respectively. In the S40b cell line, the SF_2_ for 3 and 6 days exposure was 0.43 and 0.456 and the SF_10_ was 0.00238 and 0.00165, respectively, Figure 3. Thus, the radiosensitising effect was demonstrated to be independent of the length of exposure preirradiation in both the MGHU-1 and the S40b cell lines, with exposure for 24 h having the same effect as continuous exposure for 6 days preirradiation. The radiosensitising effect was found to be independent of the dose of ZD1839 at the concentrations studied, with exposure to 10 and 0.01 *μ*M having no statistically significant difference in both cell lines (*P*=0.8 and 0.84 in MGH-U1 and S40b), Figure 4.

Both cell lines demonstrated a moderate expression of EGFR, with the radiosensitive S40b cell line expressing higher levels (21.5 fm mg^−1^ of protein) than the radioresistant MGH-U1 cell line (16.7 fm mg^−1^ of protein).

### Effects of ZD1839 on cell cycle

**Figure 5:**
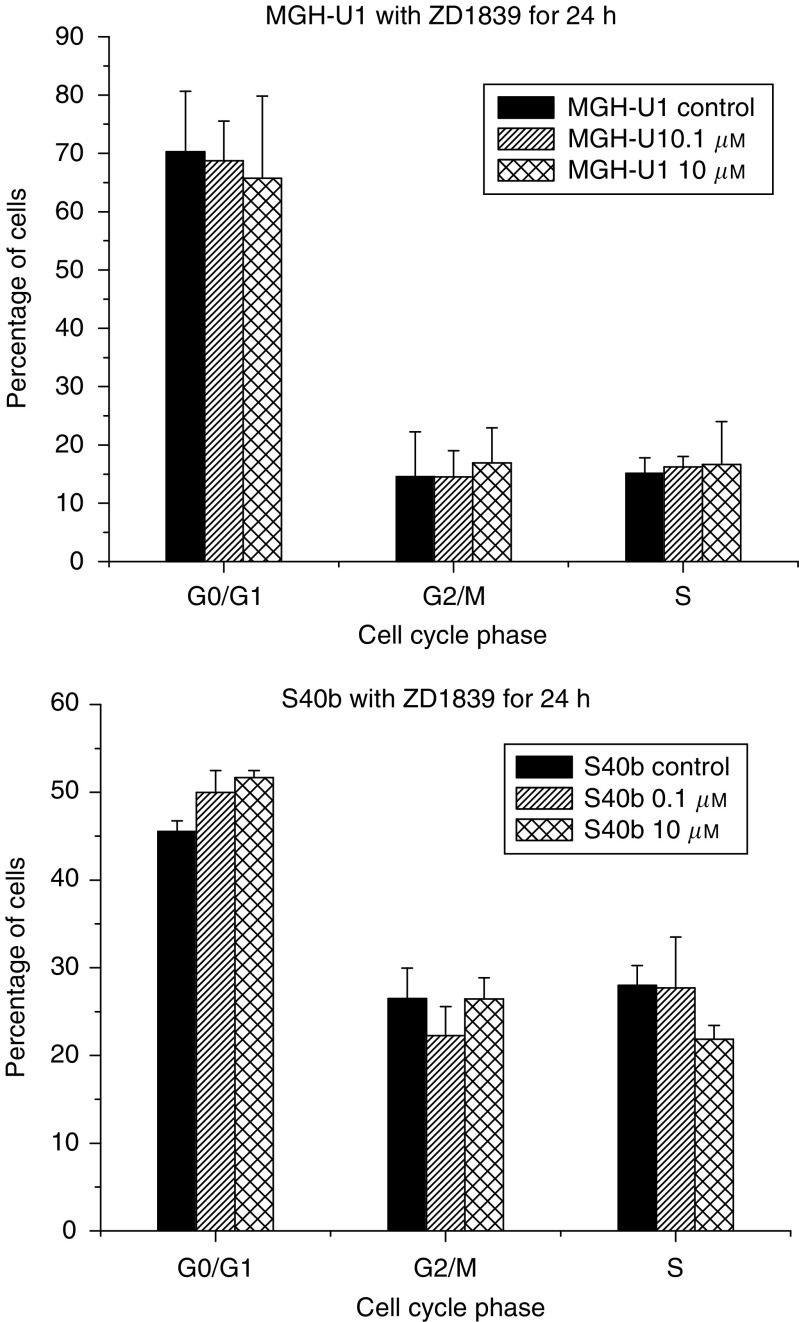
Effects of exposure to ZD1839 on cell cycle kinetics. Cells were treated with ZD1839 at concentrations of 0.1 and 10 *μ*M for 24 h. Cells were analysed 48 h after exposure using PI staining and flow cytometry. No significant difference was demonstrated in the proportion of cells in each phase of the cell cycle following exposure to ZD1839 in either cell line at either drug concentration.

Analysis of cell cycle kinetics at 0, 24 and 48 h postexposure to ZD1839 (0.1 and 10 *μ*M) revealed no demonstrable alteration in the proportion of cells in different phases of the cell cycle in relation to control cells. There was no significant accumulation of cells in the G_0_/G_1_ phase of the cell cycle in the MGH-U1 cell line (70.3% in the control sample *vs* 68.8 and 65.7% in the 0.1 and 10 *μ*M samples, respectively), or in the S40b cell line (45.5% in the control sample *vs* 49.9 and 51.7% in the 0.1 and 10 *μ*M samples, respectively). Similarly, there was no significant alteration in the proportion of cells in the S phase of the cell cycle in either cell line at either concentration of ZD1839 (15.2% in the control sample *vs* 16.2 and 16.6% in the 0.1 and 10 *μ*M samples in the MGH-U1 cell line and 28 *vs* 27.7 and 21.9% in the 0.1 and 10 *μ*M samples in the S40b cell line), Figure 5.

### Effect of ZD1839 on apoptosis

**Figure 6:**
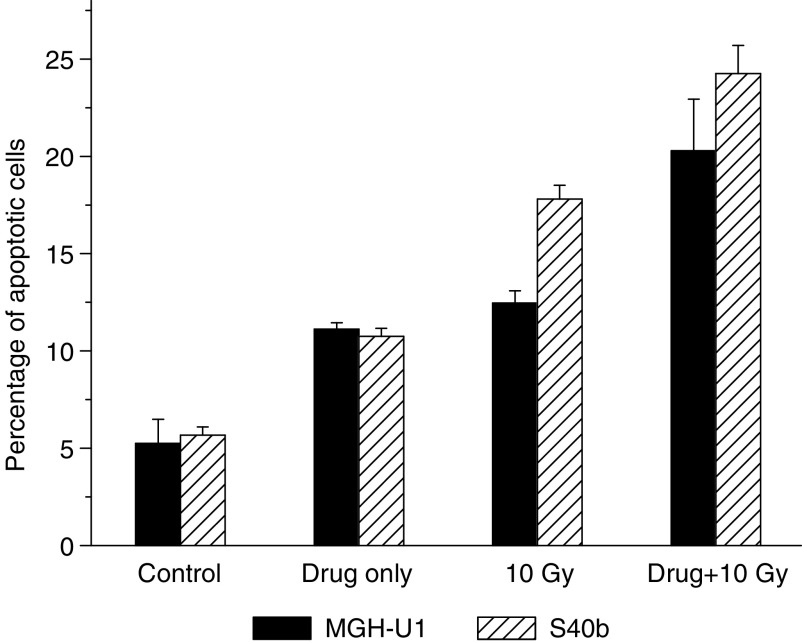
Effects of exposure to ZD1839 for 24 h on apoptosis. Cells were exposed to ZD1839 only (0.01 *μ*M), 10 Gy only or the combination of ZD1839+10 Gy. Significant induction of apoptosis was demonstrated with the combination of ZD1839+ionising radiation (*P*=0.04 for both MGH-U1 and S40b).

ZD1839 was found to have a modest proapoptotic effect on both cell lines as a single agent resulting in the induction of apoptosis in the MGH-U1 cell line (11.1% compared to control values of 5.3%). A similar effect on the S40b cell line was also noted (10.8% compared to control values of 5.7%). Treatment of both cell lines with radiation alone resulted in the induction of apoptosis in both cell lines with a greater proportionate induction in the S40b radiosensitive cell line (12.5 *vs* 5.3% in control samples for MGH-U1 cells and 17.8 *vs* 5.7% in the control sample for S40b cells). Following the combination of ZD1839 and radiation, there was a significant induction of apoptosis in both the radiosensitive and the radioresistant cell lines. In the radiosensitive S40b cell line, 24.3% of cells were undergoing apoptosis compared to control values of 5.7% (*P*=0.04), while in the MGH-U1 cell line, 20.3% of cells were undergoing apoptosis compared to control values of 5.3% (*P*=0.04), Figure 6.

## DISCUSSION

Manipulation of growth factor receptors such as EGFR and the subsequent effect on downstream signalling cascades has been of much interest in the development of novel cancer therapies (Fry, 1999). EGFR is a factor with a key regulatory role in a wide variety of solid malignancies and EGFR signalling cascades have been implicated in cellular processes such as proliferation, angiogenesis, invasion and metastasis (Salomon *et al*, 1995). Manipulation of this receptor in combination with standard cancer therapies such as chemotherapy and radiotherapy might therefore offer the potential for increased efficacy of established treatments without significantly increasing overall cellular toxicity. The two main modalities for manipulation of EGFR include the blockade of the receptor with anti-EGFR monoclonal antibodies (Herbst and Shin, 2002) and inhibition of the receptor with small molecule EGFR tyrosine kinase inhibitors such as ZD1839 (Albanell *et al*, 2001).

ZD1839 has been shown to have growth inhibitory effects across a wide variety of cell types. The IC_50_ for ZD1839 has been reported to be relatively cell line specific with values ranging from 0.15 to 31 *μ*M (Ciardiello *et al*, 2001a; Magne *et al*, 2002). In our study, ZD1839 was found to have little effect on the growth characteristics of both cell lines as a single agent when studied at the doses previously reported to have significant growth inhibitory effects (Ciardiello *et al*, 2000). This was found to be the case when cells were exposed to the drug for 24 h or up to 6 days with the drug being replenished on a daily basis. At higher concentrations, ZD1839 was found to have a growth-inhibitory effect with an IC_50_ of 98 *μ*M in the MGH-U1 cell line and 59 *μ*M in the S40b cell line. EGFR expression was found to be higher in the radiosensitive S40b cell line (21.5 fmol mg^−1^ of protein) compared to the radioresistant MGH-U1 line (16.7 fmol mg^−1^ of protein), although this does not represent particularly high expression. Previous studies demonstrating an inverse correlation between the IC_50_ of ZD1839 and EGFR expression are in agreement with our findings (Magne *et al*, 2002). However, other studies have suggested that the effects of ZD1839 on EGFR are not simply related to EGFR expression alone and may involve a complex interaction of heterodimerisation of ErbB-1 (EGFR) with other glycoprotein receptors in the subfamily such as ErbB-2 or ErbB-3 (Normanno *et al*, 2002).

One of the mechanisms by which ionising radiation has been shown to induce apoptosis is via activation of the stress-activated protein kinase pathway, also known as the c-Jun N-terminal kinase (SAPK/JNK) pathway (Verheij *et al*, 1996). However, ionising radiation can also result in the activation of EGFR which, via activation of the ras/raf mitogen-activated protein kinase (MAPK) cascade, exerts a delicate inhibitory effect upon the SAPK/JNK pathway and therefore can afford a degree of protection from the proapoptotic effects of ionising radiation (Carter *et al*, 1998; Verheij *et al*, 1998). Inhibition or an imbalance of downstream signalling cascades via an EGFR-TKI may disrupt this inbuilt protective mechanism and increase radiation-induced cell death.

ZD1839 was found to have a highly significant radiosensitising effect on both the radioresistant MHG-U1 cell line and its radiosensitive mutant clone S40b when added for 24 h preirradiation. Prolonged exposure to ZD1839 for 3 or 6 days with the drug being replenished daily did not further enhance the overall radiosensitising effect of the 24 h exposure regimen. Thus, the length of exposure to ZD1839 prior to irradiation appears to have no significant additional radiosensitising effect than exposure for 24 h preirradiation. These data would suggest that exposure to ZD1839 shortly before irradiation is sufficient to increase overall cell death and that prior treatment for greater than 24 h adds little to the radiosensitising effect. The concentration of ZD1839 was also examined to assess if this would improve the established radiosensitising effect. However, increasing the concentration of ZD1839 to 1.0 and 10 *μ*M for 24 h prior to irradiation did not increase the overall induced cell death. This suggests that the EGFR signalling cascade is sufficiently inhibited at concentrations of 0.01 *μ*M.

Exposure to ZD1839 for 24 h had no significant effect on cell cycle kinetics when analysed at 24 and 48 h postexposure. This is contrary to previously published data in a range of cell lines, where exposure has resulted in an accumulation of cells in the G_0_/G_1_ phase of the cell cycle with a subsequent decrease in the S-phase proportion (Bianco *et al*, 2002). However, our results are in agreement with published data on the mesothelioma cell line H2591, which did not display G_1_ arrest at concentrations of ZD1839 up to 10 *μ*M (Janne *et al*, 2002). This suggests that the radiosensitising effect of ZD1839 does not depend on cell cycle inhibition or alteration of cell cycle kinetics to any significant degree in the bladder cancer cell lines studied.

There was a modest induction of apoptosis with ZD1839 used as a single agent (5–6% above background) and this was similar in both cell lines. Treatment with radiation alone resulted in the induction of apoptosis in both cell lines and this was significantly more extensive for the radiosensitive S40b cell line than the parent MGH-U1 line (17.8 and 12.5%, respectively). Combination treatment with ZD1839 and radiation resulted in a marked induction of apoptosis in both cell lines compared to control cells (24.3 and 20.3% for the S40b and MGH-U1 cell lines, respectively). The mechanism by which ZD1839 promotes apoptosis as a single agent and in combination with ionising radiation is currently under investigation (Huang *et al*, 2002). Ionising radiation activates the SAPK/JNK pathway resulting in apoptosis and necrotic cell death. As previously stated, ionising radiation leads to the activation of EGFR which, via intracellular tyrosine kinase phosphorylation, results in subtle inhibition of downstream signalling messengers and reduced apoptosis. It has been suggested that ZD1839 leads to an imbalance of homo- and heterodimerisation of the ErbB family of tyrosine kinase protein receptors resulting in an increase in the EGFR-ErbB2 and EGFR-ErbB3 heterodimer complexes. These complexes are inactive resulting in a decrease of EGFR downstream signalling and loss of the EGFR inhibitory effect on the SAPK/JNK pathway resulting in an increased apoptosis (Normanno *et al*, 2001). Published studies have also proposed that ZD1839 prevents MAPK-dependent serine phosphorylation of the proapoptotic regulatory protein BAD, a member of the BH3 subfamily, resulting in it remaining in its active form (Gilmore *et al*, 2002). This inhibition of phosphorylation of serine residues also inhibits the interaction between BAD and BCL-XL, an antiapoptotic regulatory protein of the BH3 subfamily, resulting in BAD remaining in an active proapoptotic form. Reports have also suggested that ZD1839 decreases the phosphorylation of Akt via the phosphatidylinositol 3-kinase pathway resulting in decreased phosphorylation of BAD again resulting in an increase in the proportion of the active form of this regulatory protein (Kennedy *et al*, 1999). A combination of the proposed models or elements of the models may result in the loss of the fine balance of the EGFR signalling cascade, which protects against radiation-induced cell damage, thereby leading to increased cell death.

The possible mechanisms for the potentiation of radiation-induced cell death by ZD1839 remain to be established and require further investigation. However, our study suggests that despite the relatively high concentrations of ZD1839 needed to achieve cytotoxicity in bladder cancer cell lines (IC_50_ was 98 and 59 *μ*M in the MGH-U1 and S40b cell lines, respectively) and the lack of any significant alteration in cell cycle kinetics, there is a definite and pronounced radiosensitising effect in the radioresistant MGH-U1 cell line and an even greater effect in its radiosensitive mutant clone S40b. These results, in conjunction with those of a number of other groups, suggest that ZD1839 has a significant effect on potentiating radiation-induced cell death at doses higher than 2 Gy in a wide variety of cells of varying radiosensitivity. The potentiation of cell death induced in the radioresistant and the radiosensitive cell line is an encouraging feature as many tumours are inherently heterogeneous in their radiosensitivity (Steel, 1997). Radical radiotherapy is an established treatment modality for muscle invasive bladder cancer with curative results equivalent to radical surgery. This study demonstrates a possible role for ZD1839 as an adjuvant to radiotherapy as a means of overcoming radioresistance and improving outcomes in the organ sparing treatment of invasive bladder cancer.
